# Letter from Geneva

**Published:** 1884-08

**Authors:** N. W. Williams

**Affiliations:** Geneva, Switzerland


GENEVA, SWITZERLAND, June 27, 1884.

DOCTOR TAFT.—*My Dear Sir:* I am glad to see from the June DENTAL REGISTER a call for a meeting of the faculties of all the dental colleges of the United States for the purpose of adopting a uniform standard of graduation, etc. Also in the same journal that there is some talk of the dental department in the University of Michigan of extending the college term to nine months. These are steps in the right direction, and I hope the standard may be raised so high that there may be no reason given to any body to criticize our colleges as is now done in Europe. Every dentist in Europe who is a genuine American is made to blush almost every day at seeing the name of American dentist displayed in large letters on signs with unpronouncable names, and advertisements in the public prints announcing themselves as graduates of this or that dental college in the United States. We sincerely hope that something may be done whereby it will no longer be possible for any fellow who may have the courage and the money to cross the Atlantic, and after a few months sitting in the lecture room of some dental college like a wooden man, not having sufficient knowledge of the English language to make his intention or desire known to the dean of the college without the aid of an interpreter, returning to his native or some other land to swell the list already too large of those whose only claim to represent American dentistry in Europe is the possession of a diploma which they cannot read, and possessing no knowledge or ability to practice a profession of which they know nothing, that a true American is so proud of, and that is to do what he professes. It often happens that some American tourist, fresh from his native sod, is taken suddenly with the pangs of toothache, or drops a filling, or swallows an artificial tooth from his plate, and in his distress hunts up an American dentist. He sees a sign with the magic words, American dentist, and he feels happy. He enters and makes known his wants probably in this manner: "Hellow, Doc., how are you, old fellow. Have you been over long? 'Spose you are making a big fortune over here. When do you intend to return and open a broker's office on Wall street? But to proceed to business, as one would say out West, what will you charge to fill this tooth with gold, you know I won't have anything else," etc., etc., all in one breath. In the meantime the American dentist is standing with his shoulders elevated, frightened looks, and finally says in French or German that he does not understand Monsieur. Then the Yankee's turn comes to be surprised, and says, "Why, are you not an American dentist." Oui, Monsieur, or ya, Mein Herr. The American leaves the office in disgust and takes the first steamer back to America, for fear he, too, will forget how to speak English. A recent graduate from America, who has his sign out as an American dentist, and on whom an English gentleman called on a matter of business, was addressed in English, whereupon the dentist asked him to speak in French, as he did not understand English. No one need have any objection to a German, Swiss, or Frenchman holding an American degree, but how is it possible for those persons to understand the lectures in any of our colleges unless they are thoroughly acquainted with the language in which they are given? If half is true that we hear about foreigners going through our colleges so easily, there is a *screw loose* somewhere, and it ought to be tightened. It seems that our colleges are so anxious to show a long list of graduates, and especially among them many names from foreign lands, that we fear many of these applicants are allowed to pass with less trouble than one of our native boys, because the poor fellow has come so far and understands so little of the language. And then he is going so far away we will never hear if he does honor or disgrace to us. Such a state of things is fast destroying the high standing that American dentistry had taken in Europe, and if something is not done to remedy the evil, the name of American dentistry will become a reproach rather than an honor. Wishing success in every effort to elevate our specialty all over the world and with kind regards to you and yours, I am as ever, sincerely your friend,

N. W. Williams.

